# Survival Analysis and a Novel Nomogram Model for Progression-Free Survival in Patients with Prostate Cancer

**DOI:** 10.1155/2022/6358707

**Published:** 2022-03-22

**Authors:** Yuefu Han, Xingqiao Wen, Dong Chen, Xiaojuan Li, Qu Leng, Yuehui Wen, Jun Li, Weian Zhu

**Affiliations:** ^1^Department of Urology, Shenzhen Hospital, The Third College of Clinical Medicine, Southern Medical University, Shenzhen, 518100 Guangdong, China; ^2^Department of Urology, Zhujiang Hospital, Southern Medical University, Guangzhou, 510282 Guangdong, China; ^3^Department of Urology, Yuebei People's Hospital Affiliated to Medical College of Shantou University, Shaoguan, 512026 Guangdong, China; ^4^Department of Urology, Third Hospital of Sun Yat-sen University, Guangzhou, 510630 Guangdong, China; ^5^Department of Health Care, Shenzhen Hospital, The Third College of Clinical Medicine, Southern Medical University, Shenzhen, 518100 Guangdong, China; ^6^Department of Urology, Nanfang Hospital, Southern Medical University, Guangzhou, 510515 Guangdong, China

## Abstract

**Background:**

This study sought to perform a survival analysis and construct a prognostic nomogram model based on the Gleason grade, total prostate-specific antigen (tPSA), alkaline phosphate (ALP), and TNM stage in patients with prostate cancer (PCa).

**Methods:**

The progression-free survival (PFS) of 255 PCa patients was analyzed in this study. The prognostic value of tPSA and ALP was evaluated using the Kaplan-Meier survival curves and Cox regression analysis, and a nomogram model based on the Gleason grade, tPSA, ALP, and TNM stage was further established for PFS prediction in PCa patients.

**Results:**

PCa patients with different Gleason grades, tPSA and ALP levels, and TNM stages presented distinct PFS. The Gleason grade, tPSA, ALP, and TNM stage were four independent prognostic indicators. The C-index of the established nomogram was 0.705 for PFS in the test cohort and 0.687 for the validation cohort, and the calibration curves indicated a good consistency between predicted and actual PFS in PCa patients.

**Conclusion:**

The data of this study demonstrated that the Gleason grade, tPSA, ALP, and TNM stage of PCa patients are independently correlated with PFS, and a nomogram model based on these indicators may be valuable for the PFS prediction in PCa patient.

## 1. Introduction

Prostate cancer (PCa) is one of the most common malignant tumors in male. The incidence of PCa has gradually increased in recent years, which seriously threatens male health [1]. Since the lack of obvious clinical symptoms, most of PCa patients are diagnosed with advanced tumor stage, leading to the significant increase in PCa mortality rate [2, 3]. Despite the progresses in tumor therapeutic approaches, the clinical outcomes and survival prognosis of PCa remain unfavorable [4]. Therefore, it is important to early identify patients with high risk of disease progression or death, which may assist the clinical treatment and intervention in patients with PCa [5].

Prostate-specific antigen (PSA) and serum alkaline phosphate (ALP) have been identified as two critical molecular biomarkers for the occurrence and development of PCa [6, 7]. Blood PSA with a concentration of >4.0 ng/mL is an indicator for PCa screening, which has been widely used for PCa clinical diagnosis [8]. Serum ALP can be used to predict bone diseases and serves as an indicator for bone metastasis in human malignancies [9]. There are about more than 85% PCa-related deaths resulted from bone metastasis, implying the potential relationship between ALP and PCa prognosis [10]. However, there is no uniform conclusion on the role of total PSA (tPSA) and ALP in the prediction of PCa prognosis.

Nomogram is an important statistical model to predict cancer prognosis, which can easily and accurately calculate survival probability by adding multiple variables that closely associated with disease prognosis [11]. This study analyzed the relationship between clinicopathological characteristics and clinical outcomes in PCa patients and provided evidence for tPSA, ALP, Gleason grade, and TNM stage as independent indicators for PFS of PCa. A nomogram model based on tPSA, ALP, Gleason grade, and TNM stage was constructed, and their predictive value for PFS (progression-free survival) was assessed and verified in PCa patients. The established nomogram may help to predict PCa progression more intuitively and accurately and provides a basis for the optimal clinical treatment decisions.

## 2. Material and Methods

### 2.1. Patients and Sample Collection

The data analyzed in this study were collected from 255 PCa patients, who underwent therapy in the Third Hospital of Sun Yat-sen University (Guangzhou, China) and Yuebei People's Hospital (Shaoguan, China) from January 2012 to December 2018. The regular follow-up was conducted to obtain their prognosis status. Following are the inclusion and exclusion criteria for patient recruitment:

The inclusion criteria were as follows: (1) tumor tissues were histopathologically diagnosed with PCa; (2) patients had biochemical recurrence or progressed to castration-resistant PCa after ADT therapy; (3) patients were followed up regularly.

The exclusion criteria were as follows: patients suffered from other tumors, prostatitis, hepatobiliary diseases, or other conditions that might affect the detection results of tPSA and ALP.

The included PCa patients were randomly divided into test cohort (*n* = 196) and validation cohort (*n* = 59) with a ratio of 3 : 1.  Table 1 summarizes the demographic and clinicopathological characteristics of the patients, including age, history of diabetes and hypertension, bone metastasis, indwelling catheter condition, urinary tract infection, Gleason grade, TNM stage, Soloway grade, and levels of tPSA and ALP at initial diagnosis. The Gleason grades of the patients were determined with the Gleason grading system of the International Society for Urological Pathology (ISUP), the TNM stage was confirmed according to American Joint Committee on Cancer TNM 6th edition (2002), and the criteria by Soloway grade were used for different bone metastasis number. The electrochemiluminescence immunoassay by Roche cobas e8000 was used for the detection of tPSA, and the colorimetry by Roche cobas c702 methods was applied for the analysis of ALP. The protocols of this study were approved by the Ethics Committee of our organization, and a signed informed consent was provided by each participant.

### 2.2. Statistical Analysis

To facilitate the data analysis, tPSA levels were organized into 5 groups based on ≤10, 10.1-20, 20.1-50, 50.1-100, and >100 ng/mL, and ALP was divided into 4 groups by the quartile ranges (≤25%, 25.1-50%, 50.1-75%, and >75%). All the data were expressed as frequency (percentage) and analyzed using SPSS 19.0 software (IBM, Armonk, New York). The R 3.6.1. Kaplan-Meier method was used to compare the differences of PFS between groups. The univariate and multivariate Cox regression analysis was conducted to examine the effect of tPSA, ALP, and other risk factors on PFS in PCa patients. A nomogram model for predicting 1-3-year PFS of PCa patients was conducted based on the independent prognostic indicators. Harrell's concordance index (C-index) was calculated to verify the discrimination of the model. The consistency of the nomogram model using calibration curves was predicted by the internal and external validation. The results were considered statistically significant when the two-sided *P* value was less than 0.05.

## 3. Results

### 3.1. Clinicopathological Characteristic Comparison between Test and Validation PCa Cohorts

The 255 PCa patients included 196 cases in test cohort and 59 cases in validation cohort. The demographic and clinical features of the patients were recorded and compared. The results summarized in Table 1 showed that there were no statistically significant differences between the two groups in age, diabetes history, hypertension history, bone metastasis, indwelling catheter condition, urinary tract infection, Gleason grade, TNM stage, Soloway grade, and levels of tPSA and ALP (all *P* > 0.05).

### 3.2. Factors Associated with the PFS of PCa Patients

All of the clinicopathological parameters, including tPSA and ALP, were included in a Cox regression analysis to screen the factors that might be associated with the PFS of PCa patients. With the univariate analysis, the Gleason grade, tPSA, ALP, TNM stage, bone metastasis, and Soloway grade performed correlation with PFS (all *P* < 0.05, Table 2). The subsequent multivariate analysis that includes all the significant factors obtained from univariate analysis demonstrated that the Gleason grade, tPSA, ALP, and TNM stage were independently associated with the PFS of PCa patients (all *P* < 0.05). Meanwhile, the values of *P* could represent the significance of the index. Specifically, the smaller value of *P*, the higher significance is.

### 3.3. PFS in PCa Patients with Different Gleason Grades, tPSA, ALP, and TNM Stages

**Figure 1:**
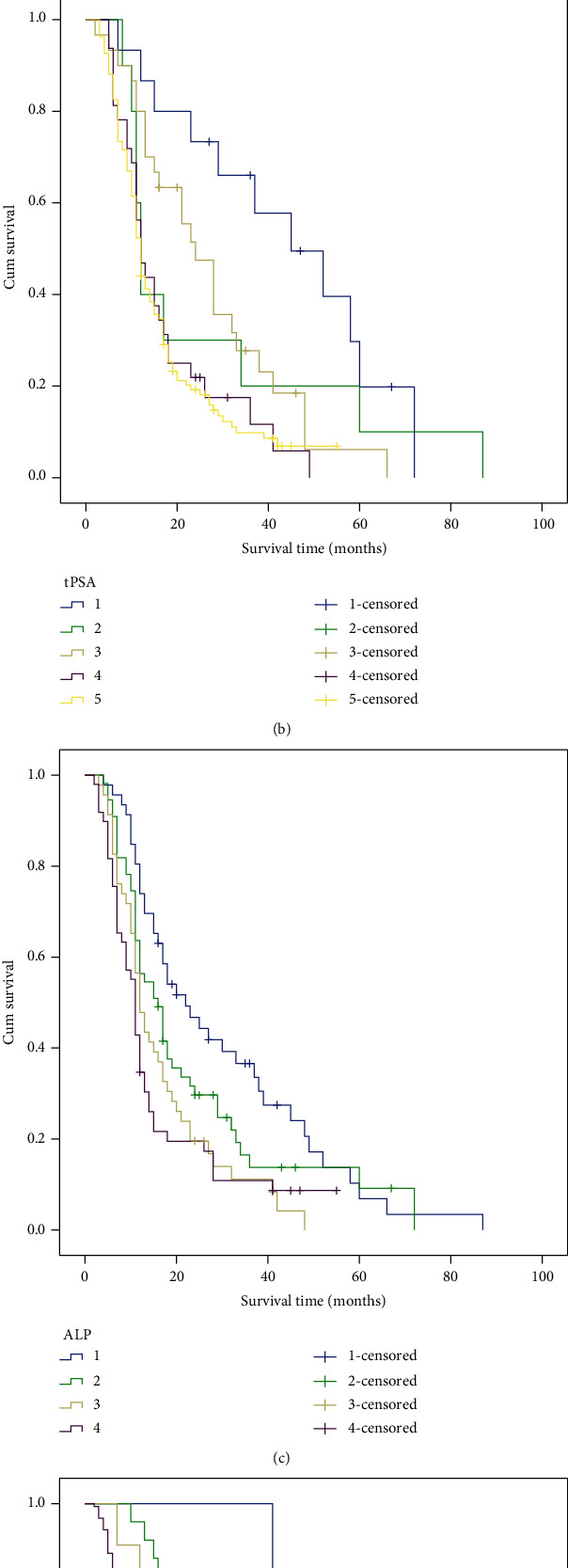
Kaplan-Meier curves for the PFS in patients with different Gleason grade, tPSA, ALP, and TNM stage. (a) Kaplan-Meier curves based on Gleason scores. (b) Kaplan-Meier curves based on tPSA levels. (c) Kaplan-Meier curves based on ALP concentration. (d) Kaplan-Meier curves based on TNM stages. ^∗^*P* < 0.05.

Given the independent association of the Gleason grade, tPSA, ALP, and TNM stage with PFS in PCa patients, the PFS in patients grouped based on these indicators was compared using Kaplan-Meier method. The Kaplan-Meier survival curves is shown in Figure 1, which indicated that PCa patients with high Gleason grade, high levels of tPSA or ALP, or advanced TNM stage had a poor PFS compared with those patients with low Gleason grade, tPSA, ALP, or early TNM stage (*P* < 0.05). In addition, the median PFS data in different groups was assessed, and the results listed in Table 3 revealed that the Gleason classification, TNM stage, and serum ALP are inversely proportional to the survival time of progression-free survival in patients with PCa. As the classification is higher, the median progression-free survival period is shorter. However, with the increase of tPSA value, the median progression-free survival of patients showed a fluctuating trend, which may be due to the influence of external factors on tPSA value.

### 3.4. Establishment of a Prognostic Nomogram Model for PFS in PCa Patients

**Figure 2:**
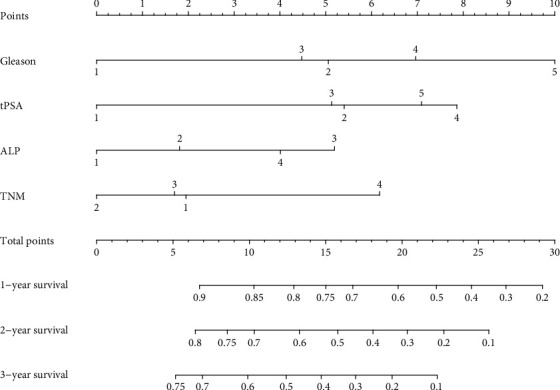
Nomogram based on the Gleason grade, tPSA, ALP, and TNM stage for predicting 1-3-year PFS in PCa patients.

A nomogram model was constructed using the Gleason grade, tPSA, ALP, and TNM stage, which were identified as independent prognostic factors of PFS after the multivariate Cox regression analysis (Figure 2). The results showed that TNM stage contributed most to PFS, followed by the Gleason grade, tPSA, and ALP. The likelihood of survival of PCa patients could be calculated by adding the scores of each variable, and the total score range was 0-30. The 1-year PFS of PCa patients accounted 0.9-0.2 when the total score was 6 to 29, and the 2-year and 3-year PFS could also be predicted by the constructed nomogram.  Table 4 lists the risk scores of the subgroups of each independent variable included in the nomogram model. The 1-3-year PFS could be predicted easily by summing up the scores of the Gleason grade, tPSA, ALP, and TNM stage for each PCa patients.

### 3.5. Nomogram Validation

**Figure 3:**
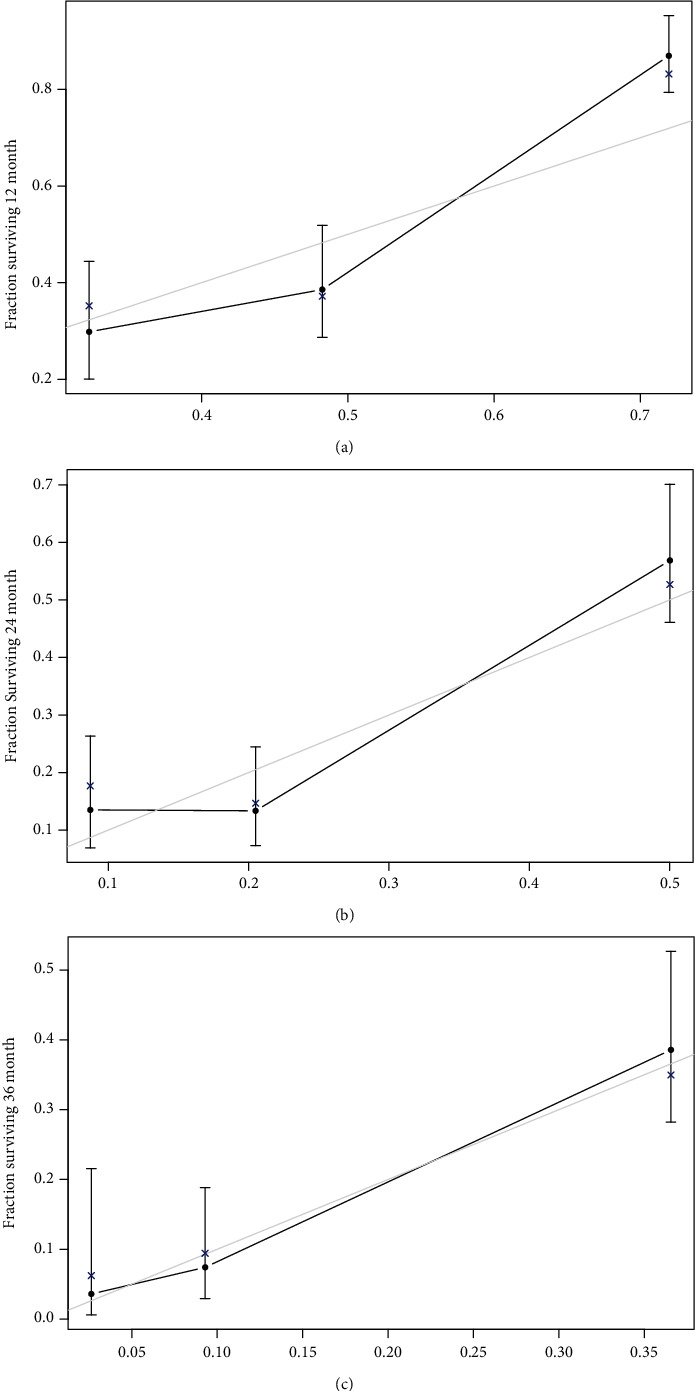
Internal calibration curves of the nomogram for predicting 1-3-year PFS in PCa patients. (a) Internal calibration curves for 1-year PFS. (b) Internal calibration curves for 2-year PFS. (c) Internal calibration curves for 3-year PFS.

**Figure 4:**
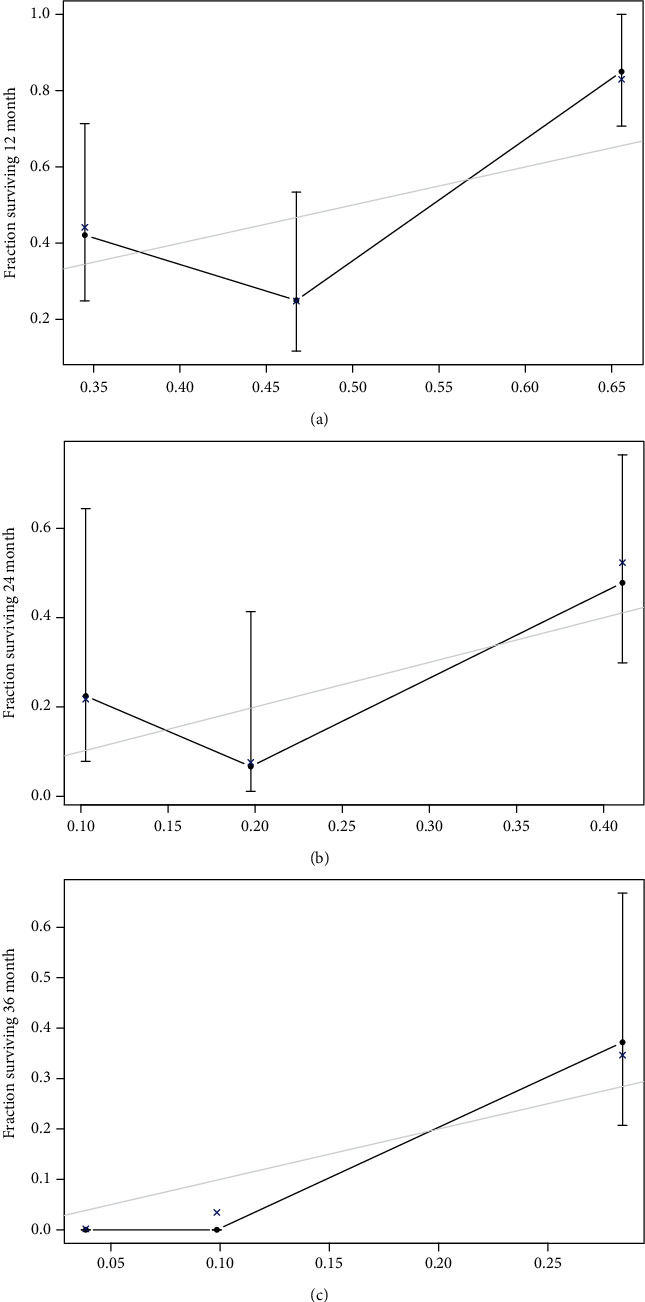
External calibration curves of the nomogram for predicting 1-3-year PFS in PCa patients. (a) External calibration curves for 1-year PFS. (b) External calibration curves for 2-year PFS. (c) External calibration curves for 3-year PFS.

In order to further formalize the validity of the nomogram, this study used data from the test set for internal verification. The results showed that C-index (95% CI) was 0.705 (0.699, 0.711), suggesting a good discrimination. The consistency test results shown in Figure 3 indicated that the predicted 1-3-year PFS was in excellent agreement with the actual PFS in the PCa patients from test set. Moreover, the C-index obtained by external validation in patients from validation test was 0.687 (95% CI of 0.664, 0.710), indicating that the discrimination was within limits of acceptability. The calibration curves shown in Figure 4 revealed that the predicted 1-3-year PFS in validation cohort was slightly lower than that in the test cohort but still presents a considerable agreement with the actual observation.

## 4. Discussion

PCa remains the most frequent malignant tumor occurred in males. This study analyzed the relationship between clinicopathological characteristics and PFS in PCa patients, aiming to screen the variables that independently associated with PFS. The Gleason grade, tPSA, ALP, and TNM stage were demonstrated to be four independent prognostic indicators for PFS prediction in PCa patients. Furthermore, a prognostic nomogram was constructed based on the identified variables, which could assist the prediction of 1-3-year PFS and showed good discrimination in the validation from both internal and external levels. In addition, the calibration curves revealed that the nomogram model could predict 1-3-year PFS accurately.

PSA is widely used for clinical screening of prostate diseases, which greatly improves the early diagnosis of PCa [12, 13]. During the development of prostate diseases, PSA levels are significantly elevated and associated with the disease progression [14]. Likewise, this study also observed that the PCa patients with high PSA levels had a poor PFS compared with those low PSA cases. Patients with PSA levels of 4-10 ng/mL are considered with benign prostate hyperplasia, and those with ≥10 ng/mL of PSA are considered with high risk of PCa. However, some PCa cases also show PSA levels of less than 10 ng/mL, leading to the application limitation of PSA [15]. Among the PCa patients included in this study, there were 15 cases with tPSA ≤10 ng/mL, accounting 5.9% of all the 255 PCa patients. Previous evidence and the survival analysis results of this study demonstrated the correlation of elevated PSA with the reduced survival in patients with PCa [16]. Nevertheless, PSA as a detection index for PCa lacks of accuracy, owing that PSA is a detection index for prostate rather than PCa [17]. It is considered that prostate infection, inflammation, or benign prostatic hyperplasia can also lead to the fluctuations of PSA levels [18]. In this study, the median PFS results in patients with different levels of PSA supported this view. Therefore, as an important indicator for PCa diagnosis and prognosis, the clinical use of PSA urgently needs to be improved.

ALP is important to indicate osteoblastic activity, which can be detected from the liver, kidney, intestinal mucosa, and bone tissues [19]. It is determined to be a predictive biomarker for tumor metastasis, especially for the metastasis to the bone [20]. There are approximately 85% PCa-related deaths caused by bone metastasis, implying the poor prognosis of PCa cases with positive bone metastasis [21]. In PCa patients, the serum upregulation of ALP has been documented to possess high predictive value for the occurrence of bone metastasis [22]. Thus, as the close relationship with bone metastasis, high levels of ALP generally predict a poor prognosis in PCa patients. In this study, ALP levels were found to be independently associated with the PFS of PCa patients, and the median PFS was reduced as the ALP concentration increases. However, a study by Wei et al. reported that ALP only increased significantly after extensive bone metastasis with limited sensitivity, and its clinical use for prognosis prediction should be performed by the combination with other parameters [7].

Currently, the Gleason grade and TNM stage are two critical references for prognosis prediction in PCa patients [23]. This study also demonstrated the independent association of the Gleason grade and TNM stage with the PFS of PCa. There are five grades (grades 1-5) in the Gleason grading system and four stages (stages I, II, III, and IV) in the TNM staging system. However, the clinical decision for PCa management based only on the Gleason grade or TNM stage maybe ambiguous and has to be confirmed by some clinical experiences. Thus, the more intuitively and accurately prognosis predictive methods are urgently needed. Nomogram as a statistical model has been applied in the prognosis prediction in various human malignancies [24, 25]. It can accurately calculate the survival for each patient by summing up multiple variables that are related with prognosis. In PCa, Brockman et al. have established a nomogram predicting model for the mortality in PCa patients with biochemical recurrence after radical prostatectomy [26]. Hou et al. have developed a prognostic nomogram to predict bone metastasis in PCa patients according to the date from SEER database [27]. The nomograms efficiently assist the clinicians to predict the clinical outcomes of patients by assessing their individualized parameters.

Considering the pivotal role of PSA and ALP in PCa development, the two indicators were included in the survival analysis, and the Gleason grade, tPSA, ALP, and TNM stage were confirmed as four important prognostic indicators by multivariate analysis. Subsequently, a nomogram model was constructed based on the four selected variables. According to the scores in the nomogram, it is easy to predict the 1-3-year PFS of PCa patients by calculating the scores of the Gleason grade, tPSA, ALP, and TNM stage. In addition, by evaluating the survival data in the test and validation sets, we confirmed the discrimination and the predictive accuracy of the nomogram model. To our knowledge, this is the first time to develop a prognosis predictive nomogram considering PSA and ALP levels in PCa patients. The prediction of PFS may be more easy and accurate with the help of this predictive nomogram model. However, several limitations are included in this study. First, the sample size is small, which may limit the identification of significant prognostic indicators. Second, some critical clinical features are missing in this study, such as smoking history, alcohol abuse, and therapy. Thus, further investigations are necessary with a larger cohort and more complete clinicopathological data. Additionally, it is necessary to establish a regression equation, which could summarize the specific role of each factor in the prediction of PFS, which should attract special attention in the future studies.

The Gleason grade, tPSA, ALP, and TNM stage are four independent prognostic factors for the PFS of PCa patients, which are used to construct a predictive nomogram model. The established nomogram can accurately predict the 1-3-year PFS of PCa with a good discrimination. In clinical practice, the nomogram model may predict individualized survival risk and guild adjuvant therapy decisions for PCa patients.

## Figures and Tables

**Table 1:** Comparison of baseline characteristics of PCa patients between test set and validation set.

Features	Test set (*n* = 196)	Validation set (*n* = 59)	*χ* ^2^	*P* value
Age, *n* (%)			3.098	0.212
≤60 years	19 (9.7)	10 (16.9)		
60-70 years	50 (25.5)	17 (28.8)		
>70 years	127 (64.8)	32 (54.2)		
History of diabetes			0.624	0.430
Yes	20 (10.2)	4 (6.8)		
No	176 (89.8)	55 (93.2)		
History of hypertension			0.034	0.854
Yes	41 (20.9)	13 (22.0)		
No	155 (79.1)	46 (78.0)		
Bone metastasis			2.682	0.101
Yes	146 (74.5)	50 (84.7)		
No	50 (25.5)	9 (15.3)		
Indwelling catheter			0.002	0.968
Yes	47 (24.0)	14 (23.7)		
No	149 (76.0)	45 (76.3)		
Urinary tract infection			1.094	0.295
Yes	23 (11.7)	10 (16.9)		
No	173 (88.3)	49 (83.1)		
Gleason grade			7.130	0.129
1	3 (1.5)	0 (0.0)		
2	22 (11.2)	4 (6.8)		
3	37 (18.9)	6 (10.2)		
4	75 (38.3)	22 (37.5)		
5	59 (30.1)	27 (45.8)		
TNM stage			5.477	0.140
I	4 (2.0)	0 (0.0)		
II	25 (12.8)	3 (5.1)		
III	11 (5.6)	4 (6.8)		
VI	156 (79.6)	52 (88.1)		
Soloway grade			4.660	0.198
0	47 (24.0)	8 (13.6)		
I	29 (14.8)	7 (11.9)		
II	25 (12.8)	12 (20.3)		
III	95 (48.5)	32 (54.2)		
tPSA (ng/mL)			8.897	0.064
≤10	15 (7.7)	0 (0.0)		
10.1-20	10 (5.1)	5 (8.5)		
20.1-50	30 (15.3)	8 (13.6)		
50.1-100	32 (16.3)	5 (8.5)		
>100	109 (55.6)	41 (69.5)		
ALP (U/L)			7.149	0.067
≤67.0	46 (23.5)	19 (32.2)		
67.1-83.0	55 (28.1)	7 (11.9)		
83.1-135.0	46 (23.5)	18 (30.5)		
>135.0	49 (25.0)	15 (25.4)		

tPSA: total prostate-specific antigen; ALP: alkaline phosphate.

**Table 2:** Univariate and multivariate Cox regression analysis results.

Characteristics	Univariate analysis		Multivariate analysis	
	HR (95% CI)	*P*	HR (95% CI)	*P*
Gleason grade				
1	Reference		Reference	
2	0.686 (0.475, 0.990)	0.044	0.706 (0.480, 1.040)	0.078
3	0.503 (0.321, 0.790)	0.003	0.527 (0.334, 0.832)	0.006
4	0.354 (0.204, 0.612)	<0.001	0.557 (0.308, 1.009)	0.054
5	0.418 (0.130, 1.350)	0.145	0.314 (0.089, 1.112)	0.073
tPSA (ng/mL)				
≤10	Reference		Reference	
10.1-20	1.866 (0.768, 4.532)	0.168	1.842 (0.687, 4.935)	0.225
20.1-50	2.031 (0.983, 4.199)	0.056	1.835 (0.828, 4.065)	0.135
50.1-100	3.608 (1.746, 7.456)	0.001	2.516 (1.103, 5.738)	0.028
>100	3.854 (1.992, 7.457)	<0.001	2.322 (1.076, 5.008)	0.032
ALP (U/L)				
≤67.0	Reference		Reference	
67.1-83.0	1.348 (0.876, 2.076)	0.175	1.234 (0.795, 1.913)	0.349
83.1-135.0	1.943 (1.246, 3.031)	0.003	1.831 (1.148, 2.920)	0.011
>135.0	2.235 (1.437, 3.476)	<0.001	1.600 (1.006, 2.544)	0.047
TNM stage				
I	Reference		Reference	
II	0.239 (0.084, 0.679)	0.007	0.593 (0.296, 1.187)	0.140
III	0.342 (0.205, 0.572)	<0.001	0.483 (0.270, 0.864)	0.014
VI	0.530 (0.271, 1.038)	0.064	0.592 (0.171, 2.045)	0.407
Bone metastasis				
Yes	Reference		—	—
No	0.447 (0.308, 0.648)	<0.001	—	—
Soloway grade				
0	Reference		—	—
I	1.357 (0.792, 2.325)	0.266	—	—
II	1.868 (1.087, 3.210)	0.024	—	—
III	3.144 (2.097, 4.712)	<0.001	—	—
Age (years)				
≤60	Reference		—	—
60-70	0.966 (0.557, 1.674)	0.901	—	—
>70	1.030 (0.622, 1.703)	0.909	—	—
History of diabetes				
Yes	Reference		—	—
No	0.795 (0.474, 1.333)	0.384	—	—
History of hypertension				
Yes	Reference		—	—
No	0.827 (0.571, 1.198)	0.316	—	—
Indwelling catheter				
Yes	Reference		—	—
No	1.413 (0.973, 2.050)	0.069	—	—
Urinary tract infection				
Yes	Reference		—	—
No	1.399 (0.820, 2.188)	0.244	—	—

tPSA: total prostate-specific antigen; ALP: alkaline phosphate.

**Table 3:** Comparison of median PFS in patients with different Gleason grade, tPSA, ALP, and TNM stage.

Grouping	Median (95% CI) (months)	*χ* ^2^	*P* value
Gleason grade		19.654	0.001
1	41.0 (0.0, 85.8)		
2	34.0 (3.2, 64.8)		
3	17.0 (13.4, 20.6)		
4	15.0 (11.8, 18.2)		
5	11.0 (9.9, 12.1)		
tPSA (ng/mL)		25.082	<0.001
≤10	45.0 (22.7, 67.3)		
10.1-20	12.0 (10.5, 13.5)		
20.1-50	24.0 (17.4, 30.6)		
50.1-100	12.0 (9.2, 14.8)		
>100	12.0 (10.9, 13.1)		
ALP (U/L)		16.938	0.001
≤67.0	22.0 (13.8, 30.2)		
67.1-83.0	16.0 (11.5, 20.5)		
83.1-135.0	12.0 (9.8, 14.2)		
>135.0	11.0 (9.9, 12.1)		
TNM grade		26.078	<0.001
I	45.0 (26.4, 63.6)		
II	32.0 (17.9, 46.1)		
III	18.0 (9.9, 26.1)		
VI	12.0 (11.2, 17.8)		

tPSA: total prostate-specific antigen; ALP: alkaline phosphate.

**Table 4:** Scores of factors involved in the prediction nomogram model.

Gleason grade	Score	tPSA (ng/mL)	Score	ALP (U/L)	Score	TNM grade	Score
5	10	≤10	0	≤67.0	0	VI	6
4	7	10.1-20	5	67.1-83.0	2	III	2
3	4	20.1-50	5	83.1-135.0	5	II	0
2	5	50.1-100	8	>135.0	4	I	2
1	0	>100	7				

tPSA: total prostate-specific antigen; ALP: alkaline phosphate.

## Data Availability

Data are available on request from the authors.
